# Real-Time Digital Twin Architecture for Immersive Industrial Automation Training

**DOI:** 10.3390/s26072023

**Published:** 2026-03-24

**Authors:** Jessica S. Ortiz, Víctor H. Andaluz, Christian P. Carvajal

**Affiliations:** 1 Departamento de Eléctrica, Electrónica y Telecomunicaciones, Universidad de las Fuerzas Armadas ESPE, Sangolquí 171103, Ecuador; vhandaluz1@espe.edu.ec; 2 Centro de Investigación MIST, Facultad de Ingenierías, Universidad Tecnológica Indoamérica, Ambato 180103, Ecuador; cpcarvajal@indoamerica.edu.ec

**Keywords:** Digital Twin, real-time systems, immersive environments, industrial IoT, networked architecture, Industry 4.0

## Abstract

Industrial automation laboratories often face limitations related to restricted access to industrial equipment, safety constraints, and limited scalability for hands-on experimentation. To address these challenges, this work proposes a real-time multi-layer Digital Twin architecture integrating a physical Siemens S7-1500 PLC, an immersive Unity-based virtual environment, HMI supervision, and IoT-enabled remote monitoring within a unified communication framework. The architecture is structured into physical, digital, and integration layers, enabling modular scalability and bidirectional synchronization between the physical process and its virtual representation through Ethernet TCP/IP communication. System performance was evaluated using synchronization metrics including communication latency, jitter, deterministic timing deviation, and event synchronization accuracy. Experimental results demonstrated stable PLC–Digital Twin communication with average latencies below 15 ms and jitter below 0.5 ms, ensuring reliable real-time interaction during continuous operation. A comparative evaluation with engineering students also showed improved learning conditions, achieving high perceived usability (SUS = 86/100) and reduced cognitive workload (NASA-TLX = 34/100). These results confirm the effectiveness of the proposed architecture as a scalable platform for Industry 4.0 training environments.

## 1. Introduction

Digital Twin technologies have emerged as a key enabler of cyber–physical integration in smart industrial environments, allowing real-time interaction between physical systems and their virtual counterparts [1,2,3]. Supported by advances in industrial communication networks, immersive visualization platforms, and Industrial Internet of Things (IIoT) infrastructures, Digital Twins facilitate high-fidelity monitoring, control, and analysis of complex automation processes [4,5]. In industrial contexts, these architectures rely on deterministic communication, synchronized data exchange, and scalable hardware/software integration to ensure temporal consistency and reliable system behavior.

Beyond pedagogical considerations, Digital Twin-based architectures also address scalability and sustainability challenges in laboratory-based automation environments [6]. Traditional laboratories are often constrained by limited equipment availability, maintenance costs, and safety risks. In contrast, synchronized physical–virtual systems enable multiple users to interact with automation processes simultaneously without compromising hardware integrity or operational safety [7,8]. Moreover, Digital Twins can be continuously updated to incorporate new processes, communication protocols, or industrial standards, ensuring alignment with evolving Industry 4.0 and emerging Industry 5.0 requirements [9,10].

Recent high-impact studies have emphasized the importance of high-fidelity synchronization and deterministic communication in Digital Twin and cyber–physical systems for industrial automation. In particular, timing-related metrics such as communication latency, jitter, and deterministic timing deviation are widely adopted to characterize real-time behavior in distributed industrial IoT architectures. These metrics provide a rigorous foundation for evaluating synchronization reliability and temporal determinism in Industry 4.0-oriented Digital Twin frameworks [11].

Although several studies have explored the use of immersive environments such as Unity and other simulation platforms for educational purposes, many of these works primarily focus on virtual simulations or pedagogical interaction frameworks without direct integration with industrial control hardware. In contrast, the approach presented in this study emphasizes the real-time coupling between a physical PLC-controlled industrial process and its virtual representation. This integration enables deterministic bidirectional communication and system-level experimentation under realistic automation conditions, distinguishing the proposed architecture from educational implementations that rely exclusively on virtual environments.

Despite the growing body of research, several limitations remain insufficiently addressed. First, many Digital Twin-based training frameworks rely exclusively on simulated models and lack real-time integration with physical industrial controllers such as PLCs, limiting realism and transferability to operational environments [12,13]. Second, while immersive interaction is frequently emphasized, structured architectural descriptions of communication layers, synchronization strategies, and protocol-level integration are often underreported [14,15]. Third, evaluation approaches commonly focus either on technical validation or on user perception, without clearly distinguishing system-level synchronization performance from measurable learning-related outcomes [16,17].

These limitations highlight an important research gap in current Digital Twin applications for industrial automation training. In particular, there is still a need for architectures that combine real industrial control hardware, immersive virtual environments, and structured evaluation methodologies within a unified framework capable of supporting deterministic real-time interaction. Furthermore, existing studies often treat technical system validation and educational impact as separate aspects, limiting a comprehensive understanding of how Digital Twin technologies contribute to both system performance and learning outcomes. Addressing these challenges requires integrated approaches that simultaneously consider architectural design, real-time communication performance, and measurable educational benefits.

In addition to the limitations identified in previous studies, it is important to position the proposed architecture with respect to other Digital Twin integration approaches reported in the literature. Several Digital Twin implementations rely on OPC-UA-based communication frameworks to connect industrial devices and virtual environments, providing standardized interoperability in distributed industrial systems. Other approaches adopt MQTT-based architectures to enable lightweight PLC–Digital Twin communication, particularly in IoT-oriented applications. Likewise, some Digital Twin training environments employ immersive VR/AR technologies to enhance visualization and user interaction, whereas Unity-based implementations focus on real-time graphical simulation and flexible system integration. More recently, edge-computing and 5G-enabled Digital Twin solutions have been proposed to support low-latency diagnostics and distributed industrial monitoring.

In contrast, the architecture proposed in this work emphasizes deterministic real-time synchronization between a PLC-controlled industrial process and its virtual representation implemented in Unity, while integrating PLC control, HMI supervision, and IoT monitoring within a modular communication structure. This approach enables the simultaneous evaluation of system-level performance and learning outcomes in industrial automation training scenarios.

To address these challenges, this work proposes a real-time networked Digital Twin architecture for industrial automation that integrates a physical PLC, an immersive Unity-based virtual environment, HMI supervision, and IoT connectivity within a multi-layer framework. The architecture enables bidirectional synchronization between physical and digital domains, ensuring deterministic communication and high-fidelity process replication while supporting scalable interaction in smart industrial environments.

Based on the identified research gaps, this work addresses two main research objectives. Objective 1 focuses on the development and technical validation of a Digital Twin architecture capable of achieving reliable real-time synchronization between physical industrial systems and their virtual representations. Objective 2 focuses on evaluating the educational and operational benefits of the proposed Digital Twin framework through structured usability, workload, and performance metrics in industrial automation training scenarios.

The novelty and main contributions of this work can be summarized as follows:

- **(Objective 1)** The design of a modular and scalable Digital Twin architecture integrating real PLC hardware, immersive visualization, HMI supervision, and IoT-based remote monitoring under a unified communication framework.
- **(Objective 1)** A detailed description of the multi-layer physical digital integration architecture supporting deterministic real-time synchronization.
- **(Objective 1)** A comprehensive technical validation using timing-related metrics such as communication latency, jitter, deterministic timing deviation, event synchronization accuracy, and update reliability.
- **(Objective 2)** A structured evaluation distinguishing system-level performance from user-centered outcomes, including usability, cognitive workload, and operational performance indicators.
- **(Objective 2)** An analysis of scalability and transferability to alternative industrial processes, PLC platforms, and distributed training scenarios.

The remainder of this paper is organized as follows. Section 2 reviews the state of the art in Digital Twin architectures and immersive industrial environments. Section 3 presents the methodology and multi-layer system architecture. Section 4 reports implementation details and experimental results, including synchronization performance and structured evaluation outcomes. Section 5 discusses the findings in relation to existing literature. Finally, Section 6 concludes the paper and outlines future research directions.

## 2. State of the Art

Digital Twin architectures and immersive virtual environments have gained significant relevance in smart industrial and cyber–physical systems, particularly in contexts requiring real-time synchronization between physical assets and virtual replicas. Initially developed for industrial monitoring, lifecycle management, and predictive maintenance, these technologies have evolved toward multi-layer architectures integrating PLC-based control, industrial communication protocols, IoT connectivity, and immersive visualization platforms. Recent research emphasizes the importance of deterministic communication, temporal consistency, and scalable hardware/software integration to ensure reliable real-time interaction within distributed industrial environments.

Beyond operational deployment, these architectures have also been explored for structured training scenarios in engineering domains where access to industrial equipment is limited due to cost, safety, or logistical constraints. However, despite the growing body of literature, several architectural and methodological limitations remain insufficiently addressed. The present section reviews representative contributions in Digital Twin synchronization, immersive industrial environments, and PLC-integrated frameworks, identifying the technical gaps that motivate the proposed approach.

The increasing availability of immersive technologies such as 3D environments developed using platforms like Unity, Unreal Engine, and other simulation-oriented graphics engines has further expanded the applicability of Digital Twins in networked and distributed industrial settings [18,19,20]. These environments enable the interactive representation of automated industrial processes, facilitating system visualization, process monitoring, and user interaction with the operation of the system in real time [21,22]. These immersive interfaces enhance visualization, operator interaction, and system supervision while maintaining real-time synchronization with programmable logic controllers (PLCs) and industrial devices [21]. Within Industry 4.0 paradigms, such integrated architectures combine physical automation hardware, communication protocols, and digital replicas into unified multi-layer systems capable of supporting operational, analytical, and training-oriented tasks.

In recent years, various studies have shown growing interest in the application of Digital Twins within technical and professional training contexts [23]. These works highlight their ability to replicate, in real time, the behavior of physical systems in virtual environments [24]. This capability strengthens conceptual understanding and supports the development of practical competencies in control and automation [5,25]. Recent research also demonstrates that immersive environments enhanced with Digital Twins increase student motivation, improve knowledge retention, and stimulate critical thinking [26]. Likewise, the integration of PLCs, Human–Machine Interfaces (HMIs), and sensor networks into these architectures brings training scenarios closer to real industrial conditions [6,21].

### 2.1. Digital Twins in Engineering Training and Industrial Contexts

Originally conceived as digital replicas for industrial monitoring, optimization, and lifecycle management, Digital Twins have evolved into broader cyber–physical architectures that support system understanding, experimentation, and decision-making in complex automation environments. In training-oriented applications, Digital Twins enable users to interact with dynamic system representations, observe internal variables, and analyze cause–effect relationships that are often inaccessible in real industrial installations [27].

Several studies report that Digital Twins contribute to improved conceptual understanding and skills acquisition in engineering domains, particularly in manufacturing systems, automation, and process control [25,28]. However, many of these contributions conceptualize Digital Twins primarily as advanced simulation tools, without fully addressing their architectural integration with physical controllers or their structured role in supporting formal training methodologies.

### 2.2. Immersive Environments for Automation and Control Systems

Immersive environments based on virtual or augmented reality provide interactive experiences that closely resemble real industrial contexts. In automation and control scenarios, these environments allow users to explore complex processes, manipulate control parameters, and visualize system responses in real time [21,29].

The literature highlights that immersion enhances engagement, particularly when scenarios reflect authentic industrial workflows [30]. Nevertheless, immersion alone does not guarantee effective system-level understanding. Without structured interaction with real process behavior and synchronized industrial hardware, immersive environments risk functioning primarily as visualization platforms rather than as integrated cyber–physical systems.

### 2.3. Active and Experiential Learning Foundations in Engineering

Active learning is widely recognized as a cornerstone of effective engineering training. Rooted in constructivist and experiential learning theories, this paradigm emphasizes learner-centered activities, iterative experimentation, reflection, and immediate feedback [25,30]. Within this framework, users construct knowledge through direct engagement with system behavior rather than passive information consumption.

Although Digital Twins and immersive environments are frequently associated with active learning, many studies adopt the term in a generic manner, without explicitly linking system interaction to structured training models. As a result, the contribution of Digital Twin-based environments often remains implicit, limiting the ability to assess their impact beyond usability or perceived engagement.

### 2.4. Real-Time Fidelity and Synchronization as Enabling Factors

For Digital Twins to function as meaningful industrial training platforms, the fidelity of interaction between physical and virtual systems is critical. High-fidelity synchronization ensures that system responses observed in immersive environments accurately reflect real process dynamics, which is essential for developing transferable operational skills.

Recent high-impact studies emphasize the relevance of real-time communication, deterministic behavior, and precise timing analysis in cyber–physical and industrial IoT systems [7,8]. Metrics such as communication latency, jitter, and timing deviation are commonly used to characterize temporal consistency. Despite their importance, these aspects are often underreported or treated qualitatively in Digital Twin training frameworks, reducing both technical rigor and system-level credibility.

### 2.5. Limitations of Existing Digital Twin-Based Training Approaches

Although existing research demonstrates the potential of Digital Twins and immersive environments in engineering contexts, several limitations persist. Many approaches rely on fully simulated models without real-time coupling to physical industrial controllers such as PLCs. Others emphasize immersive visualization without detailed architectural descriptions of communication layers or synchronization mechanisms. Furthermore, system validation and user-centered evaluation are frequently presented without clearly distinguishing technical performance from measurable training outcomes.

These limitations highlight the need for Digital Twin-based architectures that explicitly integrate real PLC synchronization, immersive interaction, and structured evaluation methodologies capable of separating system-level validation from user performance assessment. Addressing these challenges is essential to advance Digital Twins from supportive visualization tools to robust and scalable cyber–physical training platforms within Industry 4.0 and emerging Industry 5.0 environments.

Table 1 shows that existing approaches typically emphasize either (i) immersive visualization without real PLC coupling, (ii) technical Digital Twin implementations without explicit pedagogical grounding, or (iii) educational studies with predominantly descriptive evaluation. In contrast, the proposed framework combines real PLC-based synchronization, an immersive Unity environment, HMI supervision, and IoT access, while explicitly operationalizing active learning principles and reporting both technical timing metrics and structured educational outcomes.

## 3. Methodology and System Architecture

This section describes the methodological framework and system architecture adopted to design and evaluate a Digital Twin-based educational tool for industrial automation training. The methodology combines educational design principles oriented toward active learning with a technical architecture that enables real-time interaction between physical industrial systems and their virtual representation.

*Theoretical rationale (active and competency-based learning).* The methodological approach is grounded on active learning and competency-based education, where learning outcomes are defined as demonstrable abilities (e.g., PLC programming, supervision, fault diagnosis, and control tuning) and are achieved through iterative practice under authentic constraints. In this study, the Digital Twin is not treated as a visualization add-on, but as an instructional mediator that enables (i) hands-on manipulation of control logic and parameters, (ii) immediate feedback through real-time plant–twin synchronization, and (iii) guided reflection supported by measurable system responses. This design operationalizes experiential and constructivist learning cycles by structuring each activity as: task definition → action on the system → observation of effects → analysis and adjustment → re-execution, ensuring that competency acquisition is evidenced through performance in realistic automation scenarios rather than passive content exposure.

To support experiential learning in realistic industrial contexts, the proposed methodology focuses on the digitization of two representative industrial processes controlled by PLCs. The physical and virtual behaviors of these processes are synchronized through structured data exchange mechanisms, allowing students to observe, modify, and analyze system responses in real time [14]. Moreover, similar educational Digital Twin implementations combining industrial automation platforms with immersive environments such as Unity have been reported in recent case studies, highlighting their potential for hands-on training and system-level understanding [21].

The system architecture follows a modular and scalable design that separates physical processes, virtual representations, and communication mechanisms into well defined functional layers. This separation enables independent development, maintenance, and extension of each component while preserving coherent system behavior. At an abstract level, the architecture is structured around three complementary layers: (i) a physical layer responsible for process sensing, actuation, and control execution; (ii) a digital layer that provides a high-fidelity virtual representation of the industrial process for interaction and visualization; and (iii) an integration layer that ensures real-time data exchange and synchronization between the physical and virtual domains through standardized industrial communication mechanisms.

*Justification of technology selection and scalability considerations.* The selection of hardware platforms, software tools, and communication mechanisms was guided by scalability, replicability, and alignment with common industrial automation training environments. Rather than optimizing the system for a specific proprietary configuration, the proposed framework prioritizes widely adopted industrial control and visualization platforms that are representative of real-world automation practice. This design choice facilitates replication in academic laboratories with different resource constraints and supports incremental scaling toward more complex processes, a higher number of users, or extended monitoring functionalities without requiring fundamental architectural modifications.

Finally, although the framework is instantiated in this study using specific industrial automation components and processes, the proposed methodology and architectural principles are not platform dependent. The modular layering and protocol oriented integration strategy allow the framework to be transferred to alternative PLC platforms, visualization environments, or industrial processes with minimal adaptation. This supports its application across different industrial automation courses, laboratory configurations, and educational contexts, reinforcing its generalization and transferability.

### 3.1. Integrated Digital Twin Architecture for Active Learning in Industrial Automation

**Figure 1:**
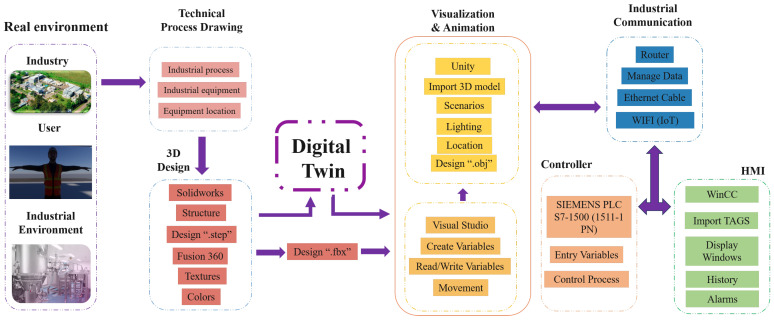
Integrated architecture of the Digital Twin system for active learning in industrial automation.

Figure 1 illustrates the integrated architecture of the Digital Twin system, representing the main stages involved in the development of the active learning tool. The physical environment includes the equipment, sensors, and controllers that make up the industrial plant. The virtual environment reproduces these elements in a three-dimensional model that replicates their dynamic behavior. The communication infrastructure connects both domains through industrial protocols, ensuring a continuous flow of data. This integration enables bidirectional synchronization between the physical process and its digital replica. As a result, the system provides a realistic, safe, and flexible learning experience aimed at developing technical competencies in industrial automation.

The system is organized in a modular way and consists of five main stages:

- *Real environment:* This stage identifies the processes, industrial equipment, and users who interact with the plant. Based on this information, technical diagrams of the process are developed, defining the layout of components and operational variables. This phase forms the foundation for the digitalization of the physical system.
- *3D design:* Modeling tools such as SolidWorks 2022 and Fusion 360 are used to create the geometric structure of the equipment, adding textures, colors, and realistic physical properties. The models are exported in standard formats (.step, .fbx, .obj), ensuring interoperability with simulation and visualization platforms.
- *Visualization and animation:* This stage is developed in Unity 3D 2021.3 LTS and Visual Studio 2019, where the 3D models are imported, and scenarios, lighting, and component motions are configured. In this environment, scripts are implemented for real-time reading and writing of variables, allowing the Digital Twin to respond to changes in the physical process and vice versa.
- *Communication:* This stage acts as the synchronization core between the Digital Twin and the physical plant. An Ethernet network managed through an industrial router connects the Siemens S7-1500 (1511-1 PN, Siemens AG, Munich, Germany) controller, which executes the control logic and manages process input and output variables. Data are transmitted to the virtual environment through standard industrial protocols. In addition, wireless (WiFi–IoT) connectivity is integrated to support remote monitoring and future expansion to distributed educational environments;
- *HMI:* Developed in WinCC Unified, the HMI module provides the main human–system interaction layer of the Digital Twin architecture. It enables real-time visualization of process variables, alarm monitoring, and supervisory control of the physical system through PLC tag integration. The HMI operates as a supervision and interaction interface, allowing users to monitor system behavior and adjust operational parameters while maintaining consistency between the physical process and its digital representation.

From an educational perspective, this integrated architecture supports active learning by allowing students to interact with industrial processes, test control strategies, and immediately observe system responses. The tight coupling between the physical system and its digital representation facilitates experiential learning through experimentation and reflection, rather than passive observation.

### 3.2. Communication and Data Synchronization Layer

The Digital Twin system establishes bidirectional communication between the physical and virtual components, ensuring synchronized operation among the Siemens S7-1500 PLC, HMI, Unity-based virtual environment, and the IoT platform. The communication follows a client–server architecture implemented through the Ethernet TCP/IP protocol, which guarantees reliable and real-time data exchange across all layers of the system. To maintain a stable and deterministic network configuration, the computer’s network interface is set to use Internet Protocol version 4 (TCP/IPv4) with static addressing, assigning fixed IP addresses to the Siemens S7-1500 PLC, the IoT2050 gateway, and the computer running Unity and WinCC Unified. This addressing scheme allows all devices to communicate efficiently within the same subnet, minimizing latency and avoiding address conflicts.

Communication between Unity and the PLC is established using the Sharp7 library, a C# wrapper of Snap7 version 1.4.2, which enables direct data exchange with Siemens S7 controllers through the native S7 communication protocol. The implementation operates over Ethernet TCP/IP with a nominal data transfer rate of up to 10 Mbit/s, allowing the virtual environment to read and write PLC memory areas and process tags in real time. To ensure deterministic behavior, process variables are exchanged using a cyclic polling mechanism with update frequencies ranging between 40 and 50 Hz, depending on the dynamics of each industrial process.

**Figure 2:**
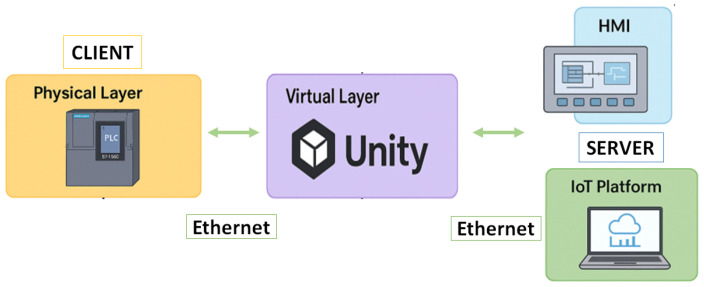
Client–Server Communication Architecture of the Digital Twin System.

Boolean, integer, and real-type PLC tags are directly mapped to corresponding variables in the Unity environment, enabling bidirectional synchronization between the physical system and its Digital Twin. Under this configuration, any change in sensor states, actuator commands, or control variables in the physical process is immediately reflected in the virtual environment, and vice versa. This mechanism ensures high-fidelity replication of process dynamics, stable real-time performance, and consistent behavior between the physical and virtual domains. The overall communication architecture integrating the PLC, Unity environment, HMI, and IoT gateway is illustrated in Figure 2.

The overall communication architecture integrating the PLC, the Unity environment, the HMI, and the IoT gateway is illustrated in Figure 2. Within this architecture, the HMI serves as the local supervision interface of the Digital Twin system and is directly connected to the PLC via Ethernet using the S7 protocol. Through this interface, users can monitor process variables, interact with system operations, and observe how changes occurring in either the physical process or the virtual environment are reflected across both domains in real time.

Meanwhile, the IoT communication layer extends system connectivity to remote monitoring platforms. The IoT2050 gateway acts as a bridge between the PLC and the Ubidots cloud via Node-RED, using the MQTT protocol for lightweight and efficient data transmission. The transmitted data are displayed on customized dashboards that show process parameters, real-time trends, and alarms, enabling remote supervision from any internet-connected device.

**Figure 3:**
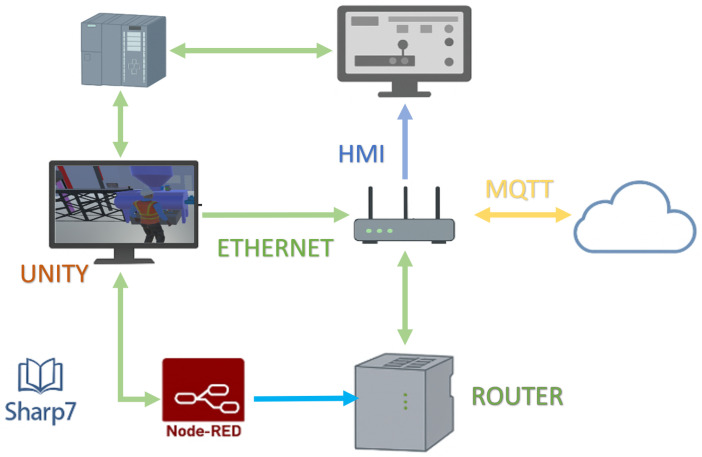
Data flow and synchronization across HMI and IoT layers in the Digital Twin system.

Building upon this integrated view, Figure 3 details the data flow and synchronization paths among WinCC Unified (HMI), the Unity-based virtual environment (via Sharp7/Snap7), the IoT2050 gateway (Node-RED), the router, and the Ubidots cloud using MQTT. This view emphasizes protocol-level interoperability (S7/Ethernet, Modbus TCP/IP where applicable, and MQTT) and the bidirectional nature of tag updates across physical and virtual layers.

### 3.3. Synchronization Performance Metrics

To quantitatively assess the real-time behavior and reliability of the PLC–Digital Twin communication, a set of synchronization performance metrics was defined and measured during continuous system operation. These metrics characterize temporal determinism, communication stability, and event-level coherence between the physical and virtual domains, providing a structured basis for the quantitative evaluation of the proposed architecture.

The selection of the synchronization performance metrics adopted in this work is consistent with recent state-of-the-art studies on real-time industrial communication and IoT-enabled cyber–physical systems. Metrics such as deterministic timing deviation and communication jitter have been extensively employed to evaluate temporal determinism, synchronization fidelity, and stability in high-fidelity Digital Twin architectures and industrial IoT frameworks.

In the proposed Digital Twin framework, reliable real-time synchronization between the physical PLC-controlled process and its virtual representation is essential to ensure consistent system behavior during monitoring, control, and training activities. Therefore, temporal determinism metrics are analyzed as part of the technical validation of the architecture.

#### 3.3.1. Temporal Determinism and Stability

Deterministic Timing Deviation (DTD) was used to quantify deviations between the nominal communication update period and the actual update intervals observed during PLC–Digital Twin data exchange. The reference update period $T_{\mathrm{ref}}$ corresponds to the nominal communication cycle configured for data exchange between the PLC and the Digital Twin environment.

Let $T_{\mathrm{ref}}$ denote this nominal update period, and $T_{i}$ represent the measured update interval at the *i*-th communication cycle. DTD is defined as:

(1) $$\mathrm{DTD}=\frac{1}{N}\sum_{i=1}^{N}\left|T_{i}-T_{\mathrm{ref}}\right|$$

where *N* is the total number of sampled communication cycles. Lower DTD values indicate higher temporal determinism and more stable real-time synchronization behavior.

Communication jitter was defined as a complementary metric to characterize short-term temporal variability in the update intervals around their mean value. It was computed as:

(2) $$J=\sqrt{\frac{1}{N}\sum_{i=1}^{N}{\left(T_{i}-\bar{T}\right)}^{2}}$$

where $\bar{T}$ denotes the mean update period computed over *N* communication cycles. This metric captures timing fluctuations introduced by network latency and processing variability.

Average Communication Latency (ACL) was defined as the mean round-trip time required for a data update to be transmitted from the PLC to the Digital Twin and back to the PLC. Latency values were obtained from timestamped message exchanges during continuous operation and averaged over *N* communication cycles.

#### 3.3.2. Event-Level Synchronization

Event Synchronization Accuracy (ESA) evaluates the consistency between discrete events generated by the PLC and their corresponding representations in the Digital Twin. Let $E_{\mathrm{PLC}}$ denote the set of discrete events generated by the PLC and $E_{\mathrm{DT}}$ the corresponding events detected in the Digital Twin. ESA is defined as:

(3) $$\mathrm{ESA}=\frac{|E_{\mathrm{PLC}}\cap E_{\mathrm{DT}}|}{|E_{\mathrm{PLC}}|}\times 100\%$$

An event was considered correctly synchronized if its corresponding Digital Twin event occurred within a predefined temporal tolerance window Δt. This metric provides an event-level measure of synchronization fidelity between the physical and virtual domains.

#### 3.3.3. Update Reliability

The Real-Time Update Success Rate (RTUSR) represents the percentage of communication cycles successfully completed within the expected timing constraints and is defined as:

(4) $$\mathrm{RTUSR}=\frac{N_{\mathrm{on}\text{-}\mathrm{time}}}{N}\times 100\%$$

where $N_{\mathrm{on}\text{-}\mathrm{time}}$ denotes the number of update cycles completed within the allowable timing window, and *N* represents the total number of communication cycles evaluated.

All timing measurements were obtained through timestamped logging at both the PLC and Unity application levels during continuous system operation. Metrics were computed over several thousand communication cycles to ensure statistical stability and repeatability under steady-state conditions.

These metrics provide a quantitative basis for validating the real-time synchronization of the Digital Twin architecture, ensuring reliable interaction between the physical and virtual systems during monitoring, control, and training activities.

### 3.4. Control Logic Implementation

**Figure 4:**
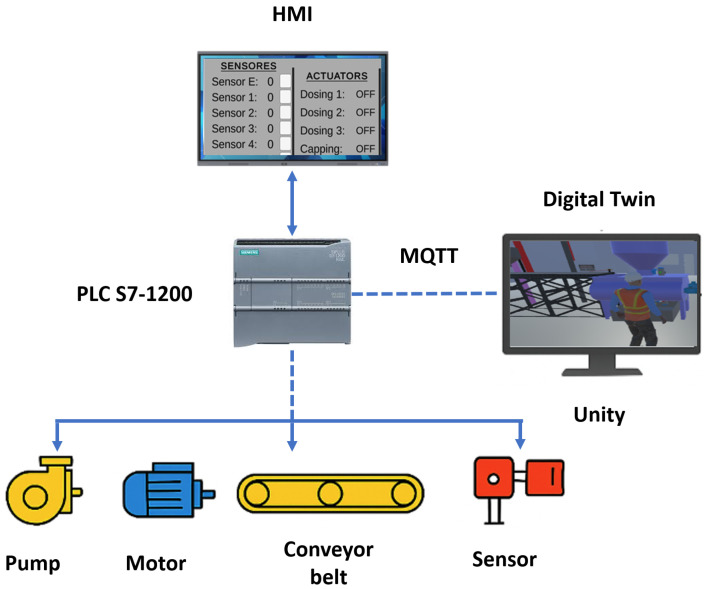
PLC–HMI–Digital Twin control architecture.

To generate the Digital Twin system, the control sequence of the physical process was defined, establishing the foundation for synchronizing the operation between the real environment and its virtual counterpart. For this purpose, two representative subprocesses of an automotive manufacturing plant were considered: the painting system and the cataphoretic electrodeposition system. The first corresponds to an automated painting process focused on the selection and mixing of primary colors to obtain secondary tones, as well as the distribution of the paint onto the product surface. This system integrates conveyor belts, presence sensors, electric actuators, motors, and dosing pumps, which realistically represent the transport, mixing, and coating application operations. The second subprocess corresponds to the cataphoretic electrodeposition system, aimed at providing a protective coating for metallic automotive structures. In this process, the control logic regulates the sequential immersion of the parts, the flow of electrical current, and the circulation of the bath fluid, ensuring a uniform deposition. Both systems share a modular control architecture designed to facilitate functional expansion, communication with physical devices, and integration with the virtual environment developed in Unity 3D, thus achieving an accurate correspondence between the real processes and their Digital Twin as illustrated in Figure 4.

The control programming is carried out in Siemens TIA Portal version 16, using ladder logic (LAD) under a sequential programming scheme. The implemented routines include system initialization, process stage enabling, safety conditions, alarm management, and proportional, integral, derivative (PID) control. The latter is used to optimize fluid dosing in pumps and speed regulation in conveyor belts, compensating for load or weight variations associated with the operating equipment. The incorporation of PID control improves the dynamic stability of the process, ensuring a smooth and precise response to disturbances or setpoint changes. In this way, a constant operational flow and an adequate level of synchronization between physical devices and their digital counterparts are maintained.

Additionally, the control system maintains bidirectional communication with the HMI, enabling real-time interaction between the user and the process. Through the HMI, critical variables can be visualized, control setpoints can be adjusted, and operational alerts can be received, promoting direct interaction with the Digital Twin and enhancing the understanding of system behavior under different operating scenarios. The programmed behavior accurately reproduces the operation of an industrial system, as the signals transmitted and received by the controller correspond to physical actuators and sensors connected to the PLC. These signals are simultaneously reflected in the Digital Twin through real-time synchronization, allowing the observation of system performance in both its physical and virtual representations.

### 3.5. System Deployment and User Interaction Workflow

The educational intervention involved a total of 32 undergraduate engineering students enrolled in the course “PLCs and Industrial Networks” of the Mechatronics Engineering program at the Universidad de las Fuerzas Armadas ESPE. All participants were in the eighth semester of the program and followed an identical curricular pathway. Prior to the intervention, the students had successfully completed foundational courses in control systems, PLC programming, and industrial automation. This shared academic background ensured a relatively homogeneous baseline in terms of prior knowledge and technical competencies among participants.

The developed learning tool is embedded in an educational framework that promotes active learning in automation and control courses for engineering students. The approach follows a competency based learning model. Students interact with didactic modules composed of a PLC, actuators, and physical sensors. These devices are mirrored in virtual environments that emulate realistic industrial processes while preserving the operating conditions and constraints of the real equipment. In this way, the virtual environment maintains the same configurations as the physical system and supports the reinforcement of theoretical concepts through practical experimentation in a digitalized work scenario.

The learning process starts with the definition of learning objectives. These objectives are aligned with core industrial automation competencies, such as PLC programming, process control, system supervision, and decision making. The educational workflow is organized into three main stages:

(i) *Process analysis.* Students identify the operating conditions and requirements of the system. Based on this analysis, they define the control logic and design the solution using functional diagrams and control sequences.
(ii) *Programming.* Once the system behavior is defined, students develop and test the control code in Siemens TIA Portal v16. After validation, the program is compiled and downloaded to the Siemens S7-1500 PLC controlling the physical laboratory module. Subsequently, the communication link with the Digital Twin developed in Unity 3D is activated through the Ethernet TCP/IP network. The physical system then generates the process events, which are transmitted to the Digital Twin and updated in real time. Students interact with the environment through the virtual interface and HMI, issuing commands that are executed by the PLC and reflected again in the Digital Twin, completing the bidirectional synchronization cycle between the physical and virtual systems.
(iii) *Supervision.* System operation is verified through the HMI, which allows students to monitor system variables and observe the behavior of the Digital Twin during operation.

Active learning within the proposed Digital Twin environment is manifested through continuous learner interaction with both the physical control system and its virtual counterpart. Students actively design control logic, modify parameters, and observe the immediate effects of their actions on process behavior in real time. This interaction loop promotes experiential learning, as learners engage in concrete experience, reflective observation, and active experimentation, in accordance with constructivist and experiential learning paradigms.

**Figure 5:**
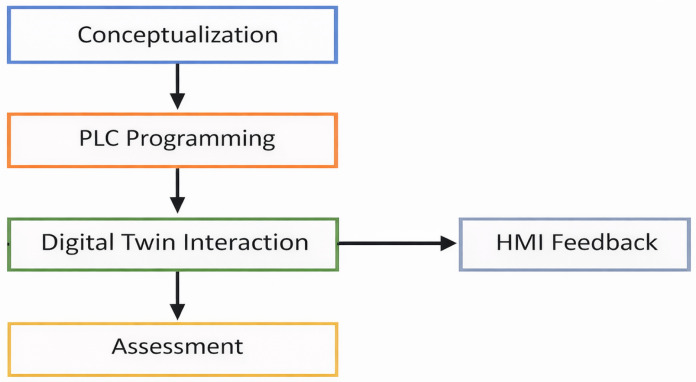
Educational workflow for PLC–HMI–Digital Twin integration.

The evaluation of learning focuses on both conceptual and procedural understanding. Students are assessed on their ability to interpret process variables, tune PID parameters, and diagnose system faults. The integration of virtual and physical experimentation promotes a hands-on learning experience while reducing risks and operational costs compared with traditional laboratory setups. Figure 5 illustrates the proposed learning workflow, from conceptual modeling to real-time interaction with the Digital Twin environment.

## 4. Results

The results reported in this section correspond to the group of undergraduate engineering students described in Section 3.5 and are framed within the educational context of the proposed Digital Twin environment. The analysis focuses on system operation, real-time synchronization performance, and educational interaction during the learning activities. Given the cohort-based nature of the study, the results are primarily examined using a descriptive and comparative approach aimed at identifying performance trends and learning-related outcomes, rather than establishing statistically generalizable inferences.

This section presents the results obtained during the implementation and validation of the Digital Twin-based system. The operational performance of the two modeled industrial processes (i) automated painting and (ii) cathodic electrodeposition was evaluated, along with the correct synchronization between the PLC, the physical environment, and the virtual replica developed in Unity 3D. In addition, the educational impact of interactive immersion, the incorporation of IoT services, and the students’ experience during system use were analyzed. Finally, usability testing and a comparison between the traditional instructional approach and the proposed interactive virtual environment are presented.

### 4.1. Industrial System Operation

This subsection reports the technical performance results of the proposed Digital Twin framework, focusing on real-time synchronization accuracy, communication latency, and system stability during continuous operation. In relation to Objective 1, which aims to ensure accurate real-time synchronization between physical industrial systems and their virtual counterparts, the following results quantify the communication reliability, temporal consistency, and operational stability achieved by the proposed framework.

The technical validation results indicate that stable communication between the PLC and the computer was achieved through the Ethernet TCP/IP protocol. This connection enabled the deployment of the control program developed in TIA Portal v16 and facilitated real-time data exchange during operation. The plant’s behavior was fully replicated in the virtual environment developed in Unity 3D, which incorporates an interactive control panel available both in its digital version and in the physical HMI of the didactic module. Proper synchronization between sensors, actuators, and 3D animations was achieved, resulting in a consistent and accurate correspondence between the physical system and its virtual representation.

Following the establishment of stable PLC–PC communication, the operational sequences of the two industrial processes the automated paint dosing system and the automotive cathodic electrodeposition process (ELPO) were successfully executed under real-time conditions. In both cases, system behavior was governed by the Ladder logic implemented in TIA Portal v16, which processed sensor inputs and triggered the corresponding physical and virtual actuators, ensuring consistent behavior between the real plant and its digital counterpart.

**Figure 6:**
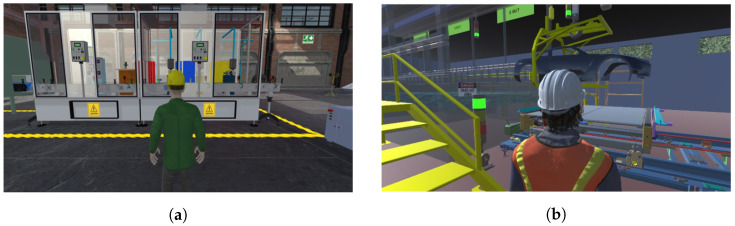
Immersive 3D virtual environments of the painting and ELPO industrial systems. (**a**) Painting process virtual environment; (**b**) ELPO process virtual environment.

Figure 6 presents immersive views generated in Unity 3D, illustrating the three-dimensional reconstruction of the painting and ELPO environments. These visualizations allow users to observe process dynamics, including material transport, dosing stages, and immersion sequences, supporting an enriched and realistic interactive learning experience.

**Figure 7:**
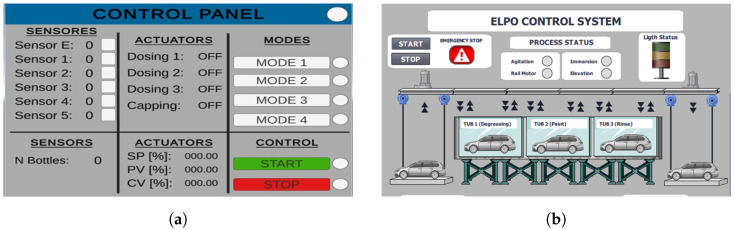
HMI interfaces developed for monitoring and control of the painting and ELPO industrial processes. (**a**) Painting process HMI interface; (**b**) ELPO process HMI interface.

Figure 7 shows the HMI interfaces developed for real-time monitoring and manual operation of both processes. The screens display system status, sensor readings, actuator states, operation modes, and critical parameters. Their integration with the PLC ensures that alarms, transitions, and process values are consistently reflected in both the virtual environment and the real instrumentation, strengthening the connection between simulation and real industrial behavior.

#### 4.1.1. Functional Testing of the Painting Process

To verify the functional behavior of the proposed Digital Twin architecture, the automated painting process was tested under different operational configurations. The evaluation focused on confirming the correct activation of sensors, dosing mechanisms, and stage transitions as represented in the virtual environment.

**Figure 8:**
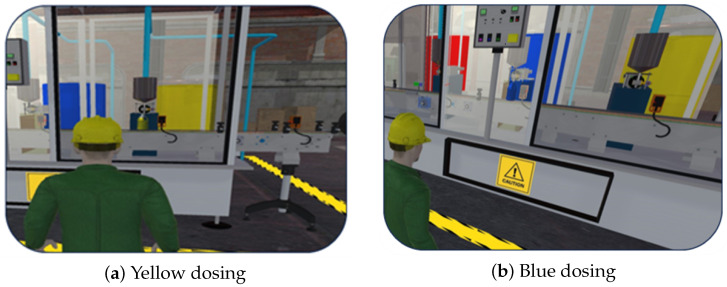
Digital Twin visualization of the initial dosing stages used for functional evaluation of the automated painting process.

**Figure 9:**
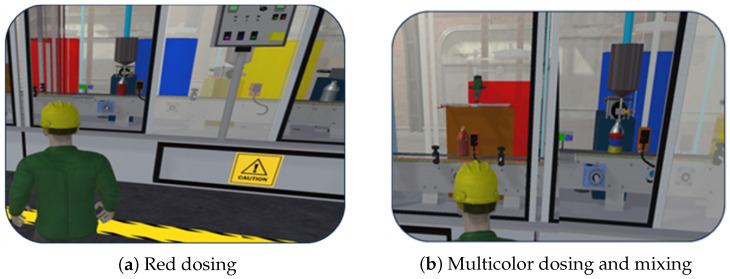
Final stages of the automated painting process, including multicolor dosing and mixing.

Figure 8 and Figure 9 illustrate four representative configurations of the process implemented in the Digital Twin environment developed in Unity 3D. These configurations include: Figure 8a yellow dosing, Figure 8b blue dosing, Figure 9a red dosing, and Figure 9b multicolor dosing followed by the mixing stage.

The visualizations allow the observation of container displacement, sensor activation, and pump operation during each stage of the process. The results confirm that the virtual environment accurately reproduces the sequence executed in the physical system, enabling visual verification of the operational states and transitions used for system evaluation.

#### 4.1.2. Functional Testing of the ELPO Process

**Figure 10:**
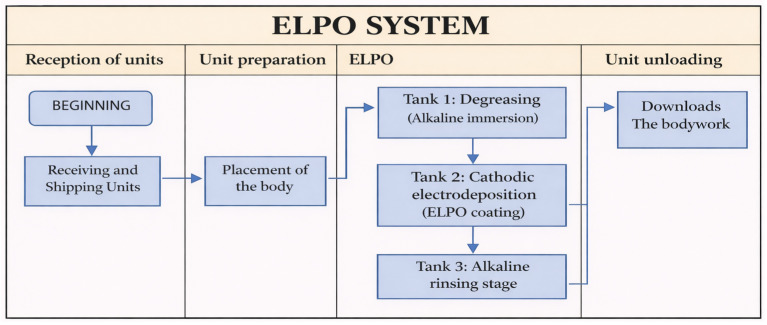
General workflow diagram of the ELPO process used as reference for functional validation. The ELPO section includes three immersion tanks: Tank 1 (alkaline degreasing), Tank 2 (cathodic electrodeposition coating), and Tank 3 (alkaline rinsing).

The functional validation of the cathodic electrodeposition (ELPO) process was conducted to verify that the PLC control program correctly responds to the signals generated by the industrial sensors and that each stage of the line operates in the intended sequence. The general workflow of the process, including body reception, preparation, immersion treatment, and unloading, is summarized in Figure 10. This diagram also served as the reference during the implementation of the Ladder logic in TIA Portal v16.

During operation, the vehicle body is sequentially transferred through three immersion tanks that perform the main stages of the ELPO treatment. Tank 1 carries out the alkaline degreasing process, removing contaminants from the metal surface. Tank 2 corresponds to the cathodic electrodeposition bath, where the protective coating is deposited through an electrochemical process. Finally, Tank 3 performs the alkaline rinsing stage to remove residual chemicals and stabilize the coating layer before unloading.

The PLC supervises digital inputs associated with position detection, immersion depth, and actuator status to ensure that each stage is executed under the correct operating conditions. Timers controlling immersion duration and oscillatory motion were also verified during the tests, since these parameters directly affect the stability of the coating process.

**Figure 11:**
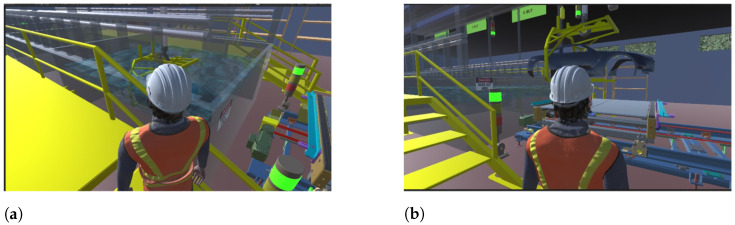
Digital Twin visualization used during the functional evaluation of the ELPO process. (**a**) Immersion dynamics in the ELPO tank; (**b**) Operator interaction in the virtual environment.

**Figure 12:**
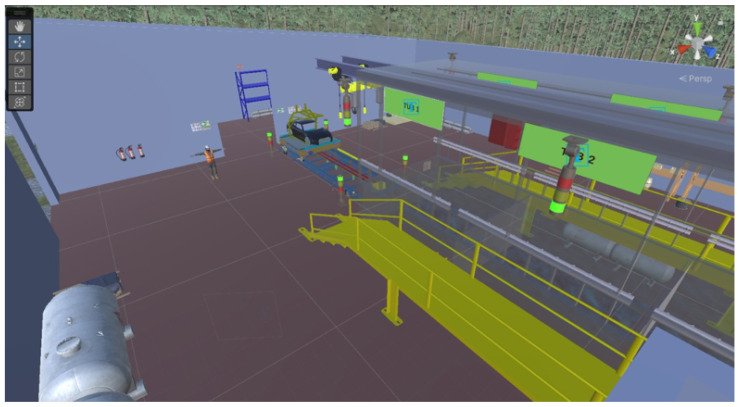
Overall view of the ELPO Digital Twin used to verify system configuration and synchronization during functional testing.

To support the evaluation, a Digital Twin of the ELPO line was developed in Unity, enabling real-time visualization of the transport mechanisms and immersion dynamics. Figure 11 illustrates the virtual representation of the process and the operator interaction with the system, while Figure 12 presents a global view of the Digital Twin environment showing the spatial configuration of tanks, walkways, sensors, and actuators. These visualizations were used to confirm the correct synchronization between the PLC control logic and the virtual process behavior during the functional testing stage.

### 4.2. PLC–Digital Twin Synchronization and Performance

The synchronization between the PLC and the Digital Twin was evaluated in the two industrial systems considered in this study: the ELPO process and the Painting process. The aim was to verify that communication between both environments remained stable, coherent in real time, and capable of supporting continuous operation. A Siemens S7-1500 controller was used as the central automation unit, taking advantage of its processing capacity and optimized communication protocols for interaction with virtual models.

Communication was established through Ethernet TCP/IP, allowing a constant exchange of process variables, sensor states, and actuator commands. This architecture enabled the Digital Twin to accurately reproduce the behavior of each system, while the PLC reliably executed the instructions generated within the virtual environment.

To understand the actual performance of this interaction, several indicators were measured, including latency, temporal variation (jitter), deterministic timing deviation, update rate, scan-cycle consistency, processor load, and event synchronization accuracy. The results obtained are summarized in Table 2.

The values show that communication remained stable at all times. Average latencies were below 15 ms, and temporal variation stayed under 0.5 ms, demonstrating a fast and predictable response. Deterministic timing deviation was also low, confirming that data synchronization occurred with a satisfactory level of precision. PLC scan-cycle consistency exceeded 98 percent, and processor load remained below 20 percent, indicating that synchronization tasks did not interfere with normal controller operation.

No packet loss or communication errors were detected during testing, confirming the integrity of the communication link. The correspondence between state changes generated in the virtual environment and the actions executed by the PLC remained above 97 percent, reflecting highly reliable behavior. This stable synchronization makes it possible to monitor the processes in real time, replicate them accurately within the virtual environment, and carry out experimentation and learning activities safely in industrial automation scenarios.

### 4.3. Educational Interaction and IoT Integration for an Immersive Experience and Remote Monitoring

The following results correspond to the educational evaluation of the proposed framework, assessing usability, cognitive workload, and student performance in comparison with a traditional laboratory-based approach. The educational evaluation metrics were selected to capture key dimensions associated with active and experiential learning in engineering education.

Addressing Objective 2, which focuses on evaluating the educational effectiveness of the Digital Twin-based approach, the following results analyze usability, cognitive workload, and learner interaction in comparison with a conventional laboratory based instructional setting.

Usability related indicators were included to assess the ease of interaction and perceived effectiveness of the Digital Twin environment, which are critical factors influencing student engagement and learning efficiency. Measures related to immersion, motivation, and interaction were incorporated to evaluate the extent to which the virtual environment supports sustained attention and meaningful engagement during practical activities. Additionally, the inclusion of remote IoT access as an evaluation dimension reflects the growing relevance of distributed monitoring and remote interaction capabilities in Industry 4.0 educational settings.

Educational experience indicators based on immersive interaction and IoT-enabled remote monitoring were evaluated across six dimensions using a five-point Likert scale (N = 32). Overall, all evaluated dimensions reported mean values above 4.0, indicating a consistently positive user perception across usability, interaction, immersion, and remote monitoring features.

Usability achieved the highest score (M = 4.7, SD = 0.4), reflecting strong user perceptions of system stability and ease of operation. This is followed by Interaction (M = 4.6, SD = 0.5) and Process Understanding (M = 4.5, SD = 0.7), suggesting that the interface and visualization mechanisms effectively supported users in exploring the simulated industrial process.

The dimensions Immersion (M = 4.4, SD = 0.6) and Motivation (M = 4.3, SD = 0.6) further support high levels of engagement during the learning activities. Meanwhile, Remote IoT Access (M = 4.2, SD = 0.7) also received a positive evaluation, although with slightly higher variability among participants.

**Figure 13:**
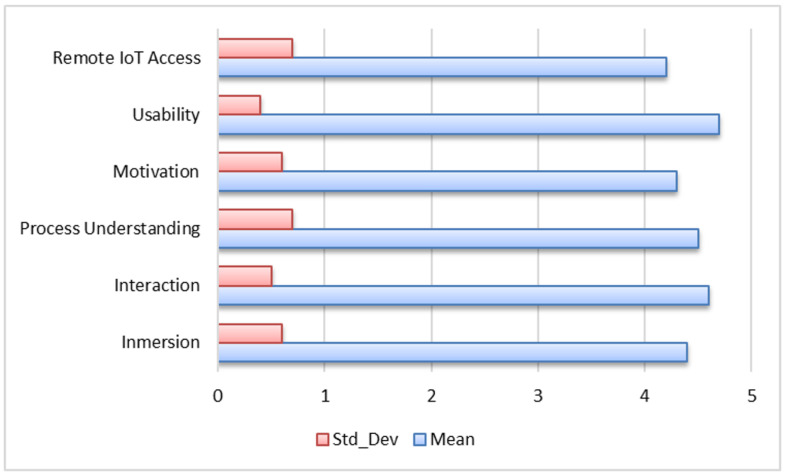
Educational experience indicators based on immersive interaction and IoT-enabled remote monitoring. Mean and standard deviation values for each evaluated dimension (N = 32).

Figure 13 provides a comparative visualization of the mean values and standard deviations across all evaluated dimensions, showing a consistent distribution of responses with relatively low variability among participants.

### 4.4. System Usability and Comparative Evaluation

This section presents a comprehensive analysis of the developed environment, integrating the evaluation of its usability, technical performance, and pedagogical effectiveness. First, the interaction of students with the Digital Twin system is examined, considering aspects such as ease of use, clarity of the interface, and the stability of synchronization with the PLC. Subsequently, the academic performance achieved through this methodology is analyzed and compared with the results obtained using traditional instructional approaches in industrial automation laboratories.

The purpose of this combined evaluation is to determine not only the functionality and robustness of the system, but also its real impact on the acquisition of operational competencies, conceptual understanding, and the level of active student engagement. The findings highlight the added value of the immersive environment and demonstrate its contribution in relation to the conventional practices employed in engineering education.

#### 4.4.1. Usability and Performance Testing

The usability and performance evaluation of the Digital Twin-based environment enabled the characterization of the quality of student–system interaction and the technical stability of the model synchronized with the PLC. During the practical sessions, students manipulated various simulated industrial processes, and objective metrics were recorded, including execution time, error frequency, operational accuracy, and virtual–physical communication latency. Additionally, the SUS and NASA-TLX questionnaires were administered to assess perceived usability and the cognitive workload associated with the tasks.

The results indicate robust system performance and a highly satisfactory user experience. The SUS score reached an average of 86/100, corresponding to an “excellent” level of usability. The cognitive workload measured with NASA-TLX was 34/100, reflecting a low-to-moderate level suitable for automation-related activities. Operationally, the average task completion time was 92 s, with a 32% reduction in operational errors and a 93% accuracy rate in the executed actions. Likewise, the average PLC–Twin communication latency remained at 78 ms, ensuring consistent responsiveness during process manipulation. Additionally, the average number of instructor interventions during the sessions was 1.2 per session, indicating a high level of autonomy among students when interacting with the system.

**Figure 14:**
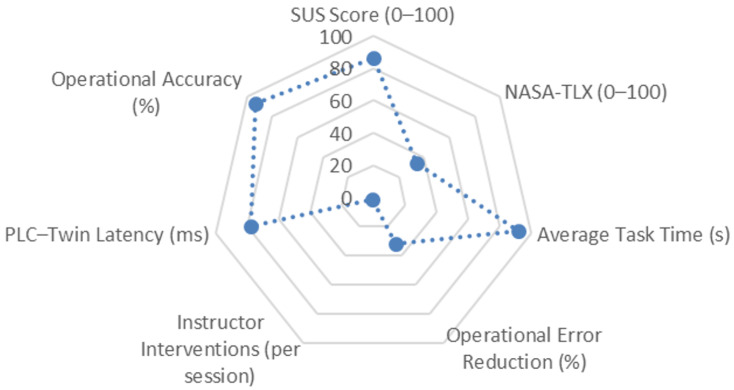
Usability and performance indicators of the Digital Twin environment.

Figure 14 summarizes the main usability and performance indicators of the Digital Twin environment. The visual representation highlights the balanced distribution among perceived usability, cognitive workload, operational accuracy, and PLC–Twin link stability, revealing a homogeneous performance profile suitable for training activities in industrial automation. Overall, the findings confirm that the Digital Twin environment provides a reliable, responsive, and pedagogically effective platform for the development of technical competencies.

#### 4.4.2. Comparison with Traditional Teaching Approaches

To evaluate the effectiveness of the proposed Digital Twin-based methodology, a comparative study was conducted with two groups of 32 engineering students each. Both groups completed the same learning tasks related to industrial automation, process control, and manipulation; however, the instructional tools differed between them. The control group worked with traditional resources, including programming software and physical didactic modules, whereas the experimental group performed the activities using the interactive Digital Twin environment synchronized with the PLC.

Both groups were composed of students from the same academic program and semester and were exposed to identical course contents and learning objectives. This configuration ensured comparable baseline conditions between the traditional and Digital Twin-based instructional approaches. Consequently, the comparative analysis focuses on identifying educational performance trends and differences in learning-related outcomes within a controlled academic context, rather than on establishing statistically generalizable conclusions.

Quantitative performance indicators were obtained through practical tests and short conceptual assessments applied at the end of each session. The analysis focused on five dimensions: operational control, conceptual understanding, active learning, error detection, and confidence in executing industrial tasks. Descriptive statistics revealed substantial differences between both instructional approaches. The traditional group achieved average scores ranging from 50% to 62%, whereas the Digital Twin group reached values between 82% and 90%. These results reflect the consistent advantage of the immersive and interactive environment over the traditional methodology.

A detailed comparison of the quantitative results highlights the performance differences between both instructional approaches. In terms of operational control, the traditional group obtained an average score of 62%, while the Digital Twin group reached 88%, representing a difference of 26 percentage points. For conceptual understanding, the traditional group achieved 58%, compared with 85% in the Digital Twin group, corresponding to a 27-point improvement.

Similarly, active learning indicators showed a notable increase, with values rising from 55% in the traditional approach to 90% in the Digital Twin-based environment, reflecting a 35-point difference. Error detection performance also improved significantly, increasing from 50% in the traditional group to 82% with the Digital Twin environment, representing a 32-point advantage. Finally, confidence in executing industrial tasks increased from 60% to 87%, corresponding to a 27-point improvement.

Furthermore, score variability was lower in the Digital Twin group, indicating more homogeneous learning outcomes. In contrast, the traditional group exhibited greater dispersion, consistent with the limited exposure to real equipment and the higher dependence on instructor guidance. These trends confirm that the Digital Twin approach provides a more stable and equitable learning experience.

**Figure 15:**
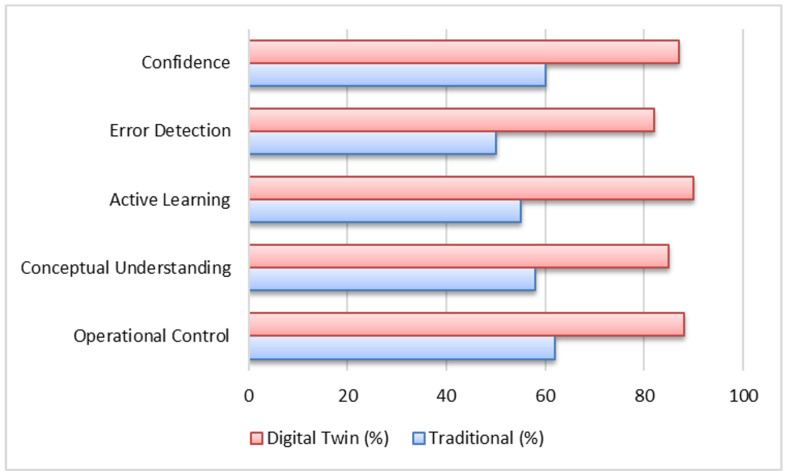
Comparative learning outcomes between traditional instruction and the Digital Twin-based methodology across five indicators.

The overall performance distributions of both groups are illustrated in Figure 15, showing consistent and marked differences across all evaluated dimensions. These findings strongly support the adoption of Digital Twins as an effective tool for enhancing conceptual understanding, operational skills, and student engagement in engineering education.

## 5. Discussion

The results obtained in this study demonstrate that the proposed Digital Twin-based educational framework effectively addresses the research objectives defined in the Introduction and constitutes a robust approach for industrial automation training. With respect to Objective 1, which focuses on achieving reliable real-time synchronization between physical industrial systems and their virtual counterparts, the observed low communication latency, minimal jitter, and high synchronization accuracy confirm that the developed architecture is suitable for real-time educational applications. Unlike purely simulated or offline virtual laboratories, the integration of an industrial-grade PLC with a Unity based Digital Twin enables faithful replication of real process dynamics, allowing learners to interact with scenarios that closely resemble real industrial operating conditions [21,33]. Similar conclusions regarding the relevance of real-time Digital Twin environments for engineering education have been reported in recent studies [39].

From an educational perspective and in relation to Objective 2, the results can be interpreted through the lens of active, constructivist, and experiential learning theories. The observed improvements in student engagement, usability perception, and task performance are not solely attributable to the technological sophistication of the Digital Twin, but rather to the way the framework enables learners to actively construct knowledge through direct interaction, experimentation, and immediate feedback [40]. By allowing students to manipulate control logic, adjust parameters, and observe the consequences of their actions on both real and virtual industrial processes, the proposed approach supports learning as an iterative, experience-driven process aligned with established principles of active learning.

From an architectural and systems perspective, the proposed framework is aligned with recent trends in industrial IoT and Digital Twin system design, which emphasize modularity, deterministic communication, and scalable integration of cyber physical components. Recent studies on high-fidelity synchronization, edge-enabled IoT architectures, and precise timing analysis highlight the growing importance of these characteristics for next-generation Industry 4.0 and emerging Industry 5.0 applications [10,29,32]. In this context, the proposed architecture, together with the selected technical and educational evaluation metrics, provides a structured and extensible foundation that can be adapted to more complex industrial systems and diverse educational scenarios.

The synchronization metrics reported in this work, including latencies below 15 ms and scan-cycle consistency above 98%, indicate that the communication infrastructure does not introduce perceptible delays or instability during operation. This level of technical stability is particularly relevant in an educational context, as it ensures that student actions are immediately reflected in both the physical and virtual domains, reinforcing the cause–effect relationships that underpin control and automation concepts. In contrast to Digital Twin learning platforms based solely on software simulation or cloud-based emulation [25,31], the proposed framework demonstrates higher determinism and responsiveness, which are critical for teaching time-sensitive industrial control tasks such as sequential logic implementation, PID, and fault diagnosis. From a pedagogical standpoint, the high usability scores and reduced perceived cognitive workload measured using standardized instruments such as SUS and NASA-TLX suggest that the immersive environment supports intuitive interaction without overwhelming learners. These findings are consistent with prior studies indicating that well-designed immersive learning environments can reduce cognitive overload while promoting deeper understanding and sustained motivation [41,42]. Moreover, the integration of real-time IoT monitoring extends the learning experience beyond the local laboratory setting, reinforcing competencies related to Industry 4.0 connectivity, data-driven supervision, and remote system analysis, as highlighted in recent literature [8].

These results are also aligned with recent research showing that Digital Twin-based learning environments can improve conceptual understanding and procedural skills in engineering education. By allowing students to experiment with industrial processes in a safe and controlled environment, such platforms promote active engagement and iterative learning through immediate feedback and system interaction. In the present study, the observed improvements in usability perception, task performance, and operational confidence are consistent with these findings, highlighting the potential of Digital Twin environments to bridge theoretical knowledge and practical industrial training.

The comparative analysis between the Digital Twin-based methodology and traditional laboratory instruction further underscores the educational value of the proposed approach. Students using the Digital Twin environment consistently outperformed those relying on conventional instructional resources across all evaluated dimensions, including operational control, active learning engagement, error detection, and procedural confidence. While similar trends have been reported in collaborative Digital Twin learning environments [21], the magnitude and consistency of the gains observed in this study can be attributed to the continuous real-time synchronization between the virtual model and the physical industrial controller, which strengthens experiential learning and procedural understanding.

Despite these positive outcomes, several limitations of the study should be acknowledged. The experimental evaluation was conducted with a single cohort of undergraduate engineering students and focused on two representative industrial processes, namely automated painting and cathodic electrodeposition. Although these processes reflect common automation scenarios, the sample size and case-study scope limit the statistical generalizability of the findings. In addition, the current implementation relies on Siemens-based automation hardware, which may constrain direct replication in environments using alternative industrial platforms. Nevertheless, the modular architecture and protocol-based communication strategy suggest that the framework can be adapted to other PLC brands and industrial control systems with minimal modifications, consistent with perspectives reported in related Digital Twin research [37,43].

In addition to the limitations associated with the experimental scope, it is also important to discuss the communication approach adopted in the implementation of the Digital Twin architecture. The proposed system relies on the Sharp7 library to establish communication with Siemens PLC controllers through the S7 protocol. While this library provides a lightweight and efficient mechanism for accessing PLC data and enabling real-time synchronization with the virtual environment, it does not incorporate some advanced capabilities typically available in industrial communication standards such as OPC UA, including standardized information models, service abstraction layers, and built-in interoperability mechanisms. Nevertheless, within the scope of the proposed educational framework, Sharp7 represents a practical and flexible solution for establishing communication between the PLC and the Digital Twin environment, enabling reliable real-time interaction during experimental validation.

Although the proposed framework demonstrated stable performance within the evaluated laboratory environment, scaling the architecture to larger industrial training scenarios may introduce additional challenges. In particular, the integration of multiple PLC-controlled processes, increased numbers of simultaneous users, and higher volumes of real-time data exchange may place greater demands on network bandwidth, synchronization mechanisms, and computational resources within the Digital Twin environment. Furthermore, extending the framework to heterogeneous industrial platforms could require additional interoperability layers to support different communication protocols and device interfaces. Addressing these challenges will require careful architectural design and potentially the incorporation of distributed or edge-computing mechanisms to maintain reliable system behavior in larger-scale deployments.

Future research will focus on extending the proposed Digital Twin environment through the incorporation of artificial intelligence techniques for adaptive learning support, automated assessment, and intelligent feedback generation. The integration of multi-user interaction, collaborative training scenarios, and predictive analytics is also envisioned to support more advanced educational use cases aligned with Industry 5.0 and human-centric automation paradigms [10,42].

## 6. Conclusions

This work presented a Digital Twin-based framework for industrial automation training that integrates real industrial control hardware, an immersive virtual environment, and real-time data synchronization. The results demonstrate that such an architecture can reliably support interactive training scenarios by enabling deterministic communication and stable real-time interaction between physical and virtual industrial processes.

From an educational perspective, the proposed framework acts as a technological enabler that supports active and experiential learning approaches. By allowing students to interact directly with realistic cyber–physical systems, the framework promotes competency-based learning, iterative problem solving, and practical understanding of industrial automation concepts. These characteristics highlight the potential of Digital Twin environments as effective tools for bridging theoretical instruction and hands-on industrial training.

Despite these promising outcomes, several limitations should be acknowledged. The experimental evaluation was conducted within a specific academic context involving a single cohort of students, a limited number of industrial case studies, and a particular automation platform. Consequently, the results should be interpreted as context-dependent and illustrative rather than universally generalizable.

Future research will focus on extending the framework to additional industrial processes, diverse automation platforms, and larger or multi-institutional student populations in order to strengthen the external validity of the results. In addition, the integration of adaptive and intelligent functionalities, such as automated feedback, collaborative learning environments, and AI-supported assessment mechanisms, will be explored to further enhance scalability and educational impact in alignment with emerging Industry 5.0 and human-centric automation paradigms.

## Figures and Tables

**Table 1:** Comparative analysis of Digital Twin-based educational frameworks in industrial automation.

Reference	Application Domain	Real PLC/ Hardware	Immersive Env. (VR/Unity)	HMI/ SCADA	IoT/ Remote	Pedagogical Grounding	Educational Evaluation	Timing/ Sync Metrics	Main Limitation/Gap
[31]	Engineering education (DT overview/ HCI)	Not explicit	Not explicit	Not explicit	Not explicit	Partial (conceptual)	Mostly descriptive/ conceptual	Not explicit	Broad scope; limited automation/PLC real-time evidence
[32]	Industrial DT (manufacturing/ CIM)	Partial	Not explicit	Partial	Partial	Not explicit	Not focus	Partial	Technical orientation; limited learning theory and classroom evaluation
[33]	DT fundamentals/ industrial systems	Not explicit	Not explicit	Not explicit	Not explicit	Not focus	Not focus	Conceptual	Reference-oriented; lacks educational experimental validation
[34]	VR simulation for learning/training	Not explicit	Yes (VR)	Not explicit	Not explicit	Partial	Usability/ engagement (varies)	Not explicit	Immersion emphasized; limited real hardware coupling
[35]	Immersive learning in education	Not explicit	Yes (VR/ immersive)	Not explicit	Not explicit	Partial (active learning)	Surveys/ perception-based	Not explicit	Educational focus; limited industrial automation realism
[26]	Industrial DT implementation	Partial/ varies	Not explicit	Partial	Partial	Not explicit	Limited	Partial	Strong technical implementation; weak pedagogical structuring
[21]	DT + industrial monitoring/ education-oriented	Partial/ varies	Partial	Partial	Yes	Partial	Mixed (often descriptive)	Partial	Needs clearer separation between technical validation and learning impact
[36]	DT for education/ sustainability framing	Not explicit	Not explicit	Not explicit	Not explicit	Partial	Mainly perception/ qualitative	Not explicit	Emphasizes benefits; limited experimental rigor and system-level evidence
[37]	Real-time DT/CPS synchronization	Yes (industrial CPS)	Not focus	Not focus	Yes	Not focus	Not focus	Yes (latency/jitter/ determinism)	Strong timing rigor; not oriented to education evaluation
[38]	Digitalization/ immersive tech (construction/ industry)	Not explicit	Partial	Not explicit	Partial	Not explicit	Not focus	Not explicit	Domain-specific; limited PLC-based automation training
**This work**	Industrial automation training (PLC–DT–HMI–IoT)	**Yes**	**Yes (Unity)**	**Yes**	**Yes**	**Explicit** **(active/** **experiential learning)**	**SUS + NASA-TLX + comparative study (n = 32)**	**Yes (latency, jitter, sync reliability)**	**Future: broader processes, larger cohorts, longitudinal learning outcomes**

**Table 2:** Synchronization performance metrics between the Siemens S7-1500 PLC and the Digital Twin for both industrial systems under real-time operation.

Metric	ELPO Process	Painting Process	Description
ACL (ms)	12.4	10.8	Average communication latency (round-trip PLC–Twin delay)
J (ms)	0.42	0.35	Communication jitter (latency variability across cycles)
DTD (ms)	1.8	1.5	Deterministic timing deviation from nominal update period
Scan-Cycle Consistency (%)	98.7	99.1	Stability of PLC scan cycle under continuous exchange
Update Frequency (Hz)	40	50	Effective Twin ↔ PLC variable exchange rate
RTUSR (%)	99.2	99.4	Real-time update success rate (on-time updates)
CPU Load During Synchronization (%)	18.5	16.9	PLC processor load during synchronization tasks
Packet Loss/CRC Errors (%)	0.00	0.00	Communication integrity during operation
ESA (%)	97.5	98.2	Event synchronization accuracy between PLC and Digital Twin

## Data Availability

The original contributions presented in this study are included in the article. Further inquiries can be directed to the corresponding author.
